# Amylin and its analogs: a friend or foe for the treatment of Alzheimer's disease?

**DOI:** 10.3389/fnagi.2014.00186

**Published:** 2014-07-29

**Authors:** Wei Qiao Qiu, Haihao Zhu

**Affiliations:** ^1^Department of Psychiatry, Boston University School of MedicineBoston, MA, USA; ^2^Department of Pharmacology and Experimental Therapeutics, Boston University School of MedicineBoston, MA, USA; ^3^Alzheimer's Disease Center, Boston University School of MedicineBoston, MA, USA

**Keywords:** amylin, amylin analogs, Alzheimer's disease, treatment, diagnosis, animal models, humans

## Abstract

Amylin, a gut-brain axis hormone, and amyloid-beta peptides (Aβ), a major component of the Alzheimer's disease (AD) brain, share several features, including similar β-sheet secondary structures, binding to the same receptor and being degraded by the same protease, insulin degrading enzyme (IDE). However, while amylin readily crosses the blood brain barrier (BBB) and mediates several activities including improving glucose metabolism, relaxing cerebrovascular structure, modulating inflammatory reaction and perhaps enhancing neural regeneration, Aβ has no known physiological functions. Thus, abundant Aβ in the AD brain could block or interfere with the binding of amylin to its receptor and hinder its functions. Recent studies using animal models for AD demonstrate that amylin and its analog reduce the AD pathology in the brain and improve cognitive impairment in AD. Given that, in addition to amyloid plaques and neurofibrillary tangles, perturbed cerebral glucose metabolism and cerebrovascular damage are the hallmarks of the AD brain, we propose that giving exogenous amylin type peptides have the potential to become a new avenue for the diagnosis and therapeutic of AD. Although amylin's property of self-aggregation may be a limitation to developing it as a therapeutic for AD, its clinical analog, pramlintide containing 3 amino acid differences from amylin, does not aggregate like human amylin, but more potently mediates amylin's activities in the brain. Pramlintide is an effective drug for diabetes with a favorable profile of safety. Thus a randomized, double-blind, placebo-controlled clinical trial should be conducted to examine the efficacy of pramlintide for AD. This review summarizes the knowledge and findings on amylin type peptides and discuss pros and cons for their potential for AD.

## Introduction

As the number of Alzheimer's disease (AD) patients grows rapidly in the U.S and globally, the need to find effective treatments for the disease becomes more urgent. Currently there are only a few medications prescribed that delay cognitive decline in AD, but their effects are modest and do not modify the underlying disease process. From the perspective of drug discovery, while it is still important to target the core pathology of AD, e.g., amyloid plaques and tauopathy in the brain, it may also be beneficial and effective to treat the downstream of the pathological cascade, including perturbed glucose metabolism, damaged cerebrovasculature, and imbalanced inflammatory reaction, which causes neuronal death and inhibit neuronal regeneration. Our recent study found that treatment with amylin, a gut-brain axis peptide, reduces the AD pathology and improves cognitive impairment in animal models for AD. This review article will summarize the research data and knowledge including our own on amylin, and propose a hypothesis that amylin class peptides can be a potential treatment for AD. Meanwhile, we will discuss the tendency of amylin to form aggregation in type 2 diabetes. The goal of this review is to debate benefit vs. harm of amylin type peptides for the treatment of AD.

### Pancreatic peptide amylin

Amylin (also known as islet amyloid polypeptide, or IAPP) is a 37–amino acid peptide hormone, and its gene is encoded on chromosome 12 and highly conserved in mammals during evolution (Nishi et al., 1989; Chang et al., 2004). While amylin is mainly produced and secreted by the β-cells in the pancreas (Westermark et al., 1987), its expression occurs in other locations such as the gut (Mulder et al., 1994) and in the sensory nervous system (Mulder et al., 1995).

Amylin peptide is stored together with insulin in dense core secretory granules in the pancreas (Lukinius et al., 1989). It is secreted in response to diet/nutrient intake (Vine et al., 1998) and exercise (Kraemer et al., 2002) stimuli, and displays a profile similar to that of insulin. The peptide circulates in a non-glycosylated (50%) and a glycosylated form (Nyholm et al., 1998), the former being the biological active compound. In healthy humans, fasting plasma amylin concentrations are in the range of 4–25 pmol/l, and amylin is distributed equally to insulin in plasma and interstitial fluids. Unlike insulin, amylin is not eliminated significantly in the liver (Kautzky-Willer et al., 1994) but mainly through renal metabolism (Hoppener et al., 2000).

Amylin belongs to the calcitonin gene peptide superfamily consisting of calcitonin (CT), calcitonin gene-related peptide (CGRP) and adrenomedullin in addition to amylin (Wimalawansa, 1997). These peptides bind to the calcitonin receptor (CTR) complexed with different receptor-activity-modifying protein (RAMPs) (Gebre-Medhin et al., 2000). As amylin readily crosses the blood brain barrier (BBB) (Banks et al., 1995; Banks and Kastin, 1998; Olsson et al., 2007), CTR and RAMPs are highly expressed in the brain. Individual RAMPs have been disrupted revealing a range of phenotypes. Mice with a disrupted RAMP1 gene were hypertensive and exhibited a dysregulated immune response, while removal of RAMP2 was lethal, and RAMP3 knockout mice appeared normal until old age when they were not as heavy as their wild-type littermates (Sexton et al., 2009). It is shown that herodimers between the CTR and RAMP1 or RAMP3 preferentially bind amylin (Christopoulos et al., 1999).

### Amylin reduces the AD pathology and improves cognitive impairment in the animal models for AD

Amyloid-β peptide (Aβ is a major component of AD pathology in the brain (Hardy and Selkoe, 2002). Amylin and Aβ share several features, including similar β-sheet secondary structures (Lim et al., 2008), binding to the same amylin receptor (Fu et al., 2012) and being degraded by Qiu et al. (1998); Bennett et al. (2003); Shen et al. (2006) or bound to insulin degrading enzyme (IDE) (de Tullio et al., 2013). Since amylin readily crosses the BBB (Banks et al., 1995; Banks and Kastin, 1998; Olsson et al., 2007), our team studied peripheral amylin's action on the amyloid pathology of AD in the brain.

**Figure 1:**
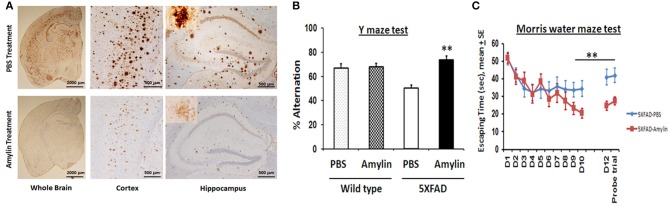
**Amylin treatment of 5XFAD mice reduces the amyloid burden and improves their learning and memory**. At 3.5 months of age, 5XFAD mice were treated by i.p. injection of PBS or amylin (200 pg/kg) daily for 10 weeks (*n* = 10 per group). **(A)** Dense-cored Aβ plaque burden is reduced in the whole brain including the cerebral cortex and the hippocampus. The amylin treated 5XFAD mice illustrated improved cognition by showing increased percent alternation in the Y maze test **(B)** (*p* = 0.001) and by showing shortened times in Morris water maze test **(C)** in finding the hidden platform at day 10 (D10) (*p* = 0.005), in memory at day 12 (D12) after the completion of training and skipping day 11 (*p* = 0.002), and in the probe trial (*p* = 0.03). Mean ± *SE* was used with ^**^p < 0.01.

Using amyloid precursor protein (APP) transgenic mice, we surprisingly found that chronic intraperitoneal (i.p.) injection of AD animals with both amylin and its analog, pramlintide, reduces the amyloid burden as well as lowers the concentrations of Aβ (Figure 1) (Zhu et al., 2014). These treatments significantly improve learning and memory in these mice as assessed by two behavioral tests, Y maze and Morris water maze. Similar to the finding by Adler et al. (2014), our unpublished data also found that elders who had mild cognitive impairment (MCI) or AD had lower concentrations of plasma amylin than controls in the absence diabetes (Table 1). Further, we found that increasing quartiles of plasma amylin were positively associated with the test scores of memory, visuospacial and executive function, but not with those of language and attention, after adjusting for demographic information, ApoE4 allele, diabetes, stroke, kidney function, and lipid profile (Table 2) (submitted and in revision). Given that impairments in these cognitive domains are signature symptoms of an early stage of AD (Weintraub et al., 2009), both mouse and human studies suggest that amylin, natural or synthetic, are likely to reduce the AD pathology in the brain and provides a new avenue of treatment for the disease.

**Table 1:** **Comparisons of plasma amylin in humans in the absence of diabetes**.

**Diagnoses**	**Controls**	**MCI**	**Alzheimer's disease**
	***N* = 104**	***N* = 82**	***N* = 26**
Median, pM/L	23.5	14.8	17.6
Q1, Q3	13.3, 47.8	8.3, 31.7	7.9, 27.9
*p*-values	–	0.01	0.05

*Median (Q1, Q3) is used to describe the distributions of plasma amylin. Using Wilcoxon rank sum test, the comparisons are shown between a disease subgroup and the controls*.

**Table 2:** **Comparisons of functions in cognitive domains across amylin quartiles**.

**Amylin quartiles**	**Quartile 1**	**Quartile 2**	**Quartile 3**	**Quartile 4**	***p*-values**
**GENERAL COGNITION**					
MMSE Scores, Mean ± *SD*	24.7 ± 4.0	25.1 ± 3.3	25.5 ± 3.3	25.3 ± 3.3	0.15
**LANGUAGE**					
Verbal fluency, Mean ± *SD*	26.9 ± 16.3	26.4 ± 12.9	28.8 ± 12.6	27.8 ± 12.0	0.17
**ATTENTION AND CONCENTRATION**					
Digit span, Mean ± *SD*	13.6 ± 3.8	13.5 ± 3.7	14.1 ± 3.6	13.9 ± 3.8	0.25
**MEMORY**					
WLL delayed recall, Mean ± *SD*	3.4 ± 2.7	3.2 ± 2.7	4.1 ± 2.9	3.8 ± 2.8	0.002
LM delayed recall, Mean ± *SD*	17.4 ± 10.2	16.8 ± 9.7	19.9 ± 9.6	19.4 ± 9.3	0.0002
**VISUOSPATIAL AND EXECUTIVE FUNCTION**					
Trailmaking A, Mean ± *SD*	98.8 ± 66.6	93.7 ± 70.3	78.2 ± 45.9	78.7 ± 53.0	<0.0001
Trailmaking B, Mean ± *SD*	223.0 ± 80.4	215.1 ± 84.1	205.0 ± 83.7	204.1 ± 86.1	0.04
Block design, Mean ± *SD*	18.7 ± 8.9	19.3 ± 8.8	20.6 ± 8.8	21.5 ± 8.7	0.004

*Mean ± SD with ANOVA test is used to describe the distributions and comparisons of test scores in each cognitive domain across the amylin quartiles. p-values for the statistical significance are shown. MMSE, Mini Mental State Exam; WLL, Word learning list; LM, Logical memory*.

**Figure 2:**
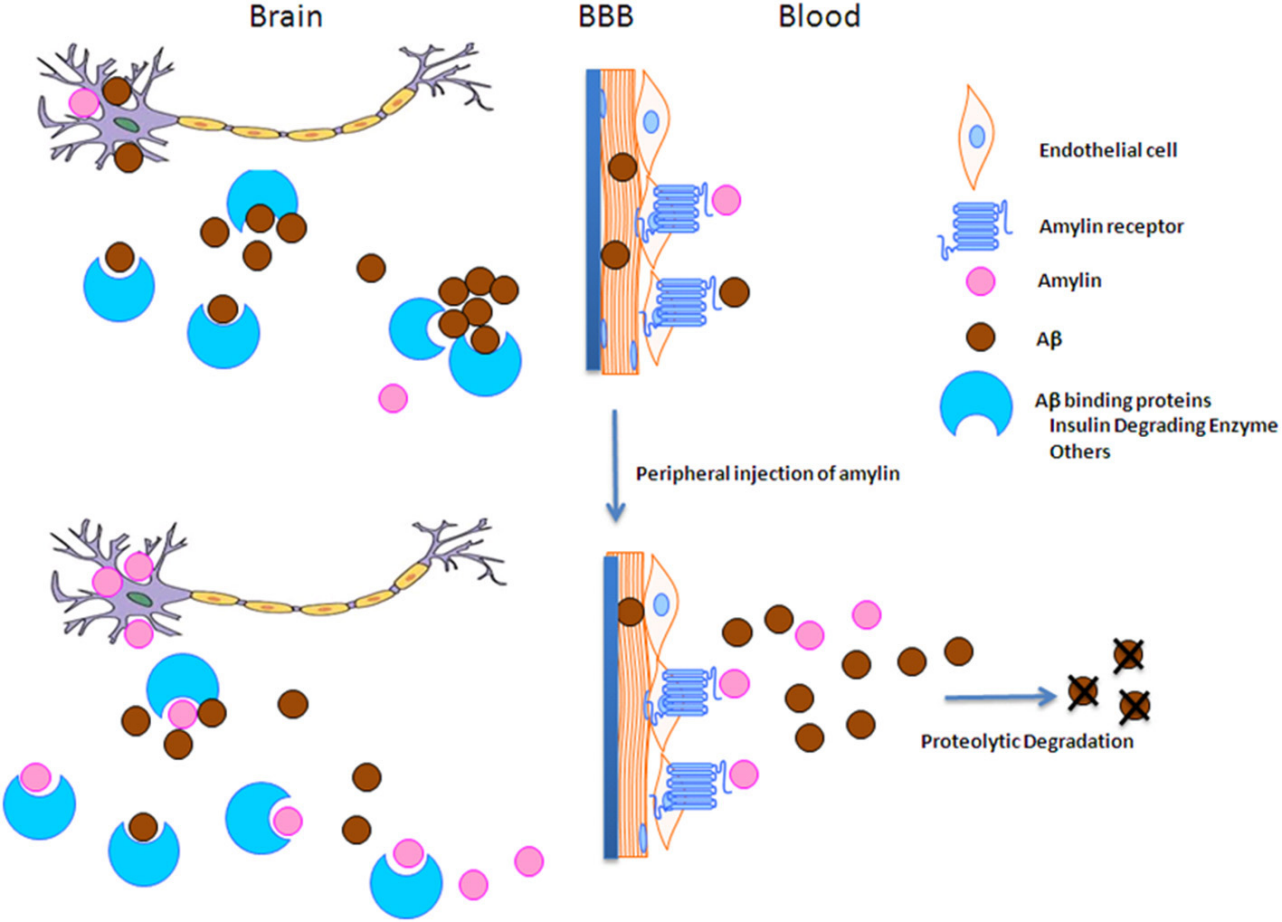
**The hypothesis of amylin type peptides as a novel therapeutic and a diagnostic tool for AD**. Since amylin and Aβ share a secondary β-sheet structure, we hypothesize that abundant Aβ in the AD brain may block amylin from binding to its receptor and hinder normal amylin functions in the brain. Thus giving extra amylin type peptides not only removes Aβ out of the brain to reduce the AD pathology in the brain, but also may restore/repair the impaired functions in the downstream of Aβ pathway in AD.

To understand how amylin type peptides improve cognition in the AD mice, our mechanistic study revealed that amylin type peptides enhance the removal of neurotoxic Aβ out of the brain. Both amylin and pramlintide treatments increase the concentrations of Aβ1-42 in cerebral spinal fluid (CSF) while reducing the amount of Aβ in the brain. A single i.p. injection of either amylin or pramlintide induces a surge of Aβ in serum, the magnitude of which is proportionate to the amount of Aβ in the brain tissue. One intracerebroventricular (i.c.v.) injection of amylin induces a more significant surge in serum Aβ than one i.p. injection of the peptide. Thus amylin treatment results in the translocation of Aβ from the brain into the CSF and blood probably through its effects on cerebral vasculature (Westfall and Curfman-Falvey, 1995; Edvinsson et al., 2001). If our mouse findings that amylin removes Aβ from the brain were relevant to humans, we would expect amylin to be positively associated with Aβ in human plasma samples. Indeed, using human fasting plasma samples, we found that concentrations of Aβ1-42 (*P* < 0.0001) and Aβ1-40 (*P* < 0.0001) increased with each quartile increase of amylin (Qiu et al., 2014) after adjusting for age, gender, ethnicity, ApoE4, BMI, diabetes, stroke, kidney function and lipid profile. Given that there is abundant Aβ in the AD brain, a stronger positive association between amylin and Aβ1-42 as well as Aβ1-40 was found in patients with AD or amnestic MCI than the one found in elderly with normal cognition (Table 3). Figure 2 shows our hypothesis that abundant Aβ, in either monomeric, oligomeric or fibriller form, in the AD brain may block the ability of amylin to bind to its receptor and interefere with normal amylin functions in the brain; giving exogenous amylin class peptides could rescue the amylin activities in the brain as well as removing Aβ out of the brain.

**Table 3:** **Correlations between Aβ and Amylin in Plasma in Humans**.

**Diagnoses**	**Controls**	**Amnestic MCI**	**Alzheimer's disease**
	***N* = 145**	***N* = 16**	***N* = 42**
Age, year, Mean ± SD^*^	72.3 ± 8.0	75.7 ± 8.7	80.5 ± 8.1
MMSE, Mean ± SD^*^	27.1 ± 2.6	26.4 ± 2.5	22.2 ± 3.3
Log10 Amylin with Log10 Aß1-42	*r* = +0.06, *p* = 0.46	*r* = +0.73, *p* = 0.001	*r* = +0.52, *p* = 0.0004
Log10 Amylin with Log10 Aß1-40	*r* = +0.02, *p* = 0.83	*r* = +0.58, *p* = 0.02	*r* = +0.29, *p* = 0.06

*Using ANOVA analysis, age and average Mini Mental State Exam (MMSE) scores in the subgroups of the controls, amnestic mild cognitive impairment (amnestic MCI) and Alzheimer's disease are compared*.

* *p < 0.0001. Pearson analyses were performed to determine correlation coefficient between plasma Aβ40 or Aβ42 and amylin in different subgroups: the controls, amnestic MCI and Alzheimer's disease. p-values for statistical significance are shown*.

Independently, Adler et al., used pramlintide to treat another AD mouse model, SAMP8, which have increased amount of Aβ and other AD pathology (Adler et al., 2014). They found that pramlintide treatment improved the performance of these mice in the novel object recognition task. Their study demonstrated that the pramlintide-treated mice had increased expression of the synaptic marker synapsin I and the kinase cyclin-dependent kinase-5 in the hippocampus, as well as decreased oxidative stress and inflammatory markers in the hippocampus. Thus the effects of amylin type peptides for AD extend beyond just the removal of Aβ from the brain, distinct from the targets of current AD drug discovery (Dunkel et al., 2012).

### Other amylin activities that are potentially beneficial for AD

Since the first description of amylin 25 years ago (Cooper et al., 1987; Westermark et al., 1987), a large body of data has documented the physiological actions of this peptide in the brain (Roth, 2013). In contrast to amylin, there is no known physiological function of Aβ in brain. Aβ in the AD brain might interfere with the following functions mediated by amylin, and thus giving extra amylin type peptides may restore/repair them.

#### Amylin improves glucose metabolism

Multiple imaging studies have demonstrated perturbed cerebral glucose metabolism in the AD brain (Johnson et al., 2012). Amylin is an important hormone regulating glucose metabolism in the body (Min et al., 2011; Roth, 2013). Since it readily crosses the BBB, and the brain accounts for approximately 50% of the body's glucose use (Bryant et al., 2002), amylin may play a similar role in the brain. Abundant Aβ in the AD brain may block or interfere with this activity of amylin.

Amylin regulates glucose metabolism in the body through three primary mechanisms in the brain, (1) slowing the rate of gastric emptying, (2) suppressing post-meal glucagon secretion and (3) reducing food intake (Roth et al., 2012; Roth, 2013). Amylin acts on the area postrema (AP), nucleus of the solitary tract (NTS), lateral parabrachial nucleus and the central nucleus of the amygdala in the brain to mediate these activities (Boyle and Lutz, 2011; Roth, 2013). Aspiration of the AP abolishes amylin's effects on gastric emptying (Young, 2005; Wickbom et al., 2008). Antagonism of amylin receptors with AC187 increases food intake and abrogates the anorexigenic effects of exogenous peripheral amylin (Mollet et al., 2004). Amylin's activity in these brain areas is mediated through inducing c-Fos expression (Rowland et al., 1997).

Amylin knock-out mice do not show any differences in basal insulin and glucose concentrations compared to wild types throughout development (Gebre-Medhin et al., 1998a), and there are also no differences in body weight, body composition or plasma leptin concentrations observed when mice are maintained on a low-fat diet (6 weeks) (Turek et al., 2010). Consistently, global deletion of the CTR has no effects on body weight or metabolic disease-related endpoints (Davey et al., 2008). Some studies reported that amylin stimulates insulin secretion (Fehmann et al., 1990), while another one demonstrated that amylin inhibits insulin secretion when the concentration of insulin is high (Gebre-Medhin et al., 2000). It should be noted that in response to changes in ambient glucose concentrations *in vivo*, amylin and insulin mRNA expressions appear to be regulated in parallel (Alam et al., 1992). Using amylin null mice (Gebre-Medhin et al., 1998a) and amylin transgenic mice (Ahren et al., 1998), it is shown that amylin plays a major role in inhibiting insulin secretion by pancreas and decreasing glucose tolerance after a glucose loading. All these researches suggest that amylin is probably not a homeostasis peptide to maintain metabolism in the body, but it is more likely a regulatory peptide responding to metabolic or other environmental stimuli.

#### Amylin promotes cell growth

It has been proposed that amylin may play a role as a trophic factor (Potes and Lutz, 2010). Amylin is shown to stimulate osteoblast growth (Cornish et al., 1998). After injection of alloxan, more severe diabetes and β cell damage/dysfunction were developed in amylin null mice than in the wild type mice (Mulder et al., 2000), probably due to the lack of amylin to promote growth and regeneration of cells after the insult (Gebre-Medhin et al., 2000). Amylin deficient mice showed reduced pain reaction when challenged with formalin, suggesting an essential role of amylin in the function of sensory neurons (Gebre-Medhin et al., 1998b; Mulder et al., 1999). Additionally, amylin infusion enhanced neurogenesis in the hippocampus and the area postrema in the brain of rodents with ovariectomy and improved their immobility in the forced swim test (Trevaskis et al., 2010). Amylin may be involved in the formation of synapsis by inducing the expression of synapsis I and cdk5 (Adler et al., 2014). The neuronal protective activity of amylin under pathological insults can be beneficial to reversing the effects of neuronal degeneration.

#### Amylin modulates inflammatory process

Human amylin has been shown to be a modulator of inflammation, especially activation of the NLRP3 inflammasome, peripherally (Masters et al., 2010). Mice with a disrupted RAMP1 gene exhibited a dysregulated immune response (Sexton et al., 2009). Amylin induces production of interleukin 1β (IL-1β) through CD36 receptor (Sheedy et al., 2013). Human amylin, but not rat amylin or Aβ, stimulates the release of the gradulocyte-macrophage colony-stimulating factor (G-MCSF) in eosinophils, and inhibits the *in vitro* interleukin-5 (IL-5)-mediated survival of eosinophils (Hom et al., 1995). After unilateral adjuvant-induced inflammation, expression of amylin is upregulated in innervating sensory neurons and is involved in the the inflammatory response (Mulder et al., 1997). In inflammatory models characterized with a vascular component including mouse ear oedema induced by croton oil and acetic acid-induced peritonitis, amylin exerts anti-inflammatory activity (Clementi et al., 1995). All these data demonstrate that amylin is a modulator in peripheral inflammation. Whether or not and how amylin modulates neuroinflammation in the brain are not yet known.

#### Amylin and Aβ mediate different intracellular signal transduction

Despite the fact that amylin and Aβ bind to the same amylin receptor (Fu et al., 2012), a recent study shows that while amylin and pramlintide increase intracellular cAMP, an important secondary messenger for learning, memory and mood, Aβ1-42 does not influence intracellular cAMP (Gingell et al., 2014). The data suggest that amylin and Aβ do not mediate the same physiological function within cells. Additionally, amylin activates the extracellular-signal regulated kinase ½ (ERK1/2) pathway by inducing the phosphorylation of ERK1/2 (pERK) (Potes et al., 2012); in contrast, Aβ decreases pERK in neurons, leading to the generation of toxic tau phosphorylation and fragments (Reifert et al., 2011). A recent study shows that amylin treatment can increase glutamate release enough to cause cell firing (Fukuda et al., 2013), which may be necessary for restoring learning and memory in AD (Danysz and Parsons, 2012).

### Amylin's self aggregation feature under pathological conditions

The amylin amyloid deposits were first found in the pancreas in diabetic patients in Opie (1901). These amylin aggregates disrupt islet structure and contribute to the β cell dysfunction in most type 2 diabetes patients (Hoppener et al., 1994; Hull et al., 2004). On the other hand, the Aβ aggregation and amyloid plaques are identified as a hallmark pathology in the AD brain and have been thought to be a key element in the AD pathogenesis (Hardy and Selkoe, 2002). Although amylin and Aβ have little or no amino acid sequence homology, the core structure of the fibrils from their amyloids is essentially the same (Sunde et al., 1997). A recent study found an accumulation of amylin amyloid in the cerebrovascular system in the AD brain (Jackson et al., 2013). It is possible that the pathological environment of the AD brain causes any amyloidgenic peptides, including amylin, to aggregate and become cell toxic.

The mechanism(s) responsible for amylin amyloid formation in type 2 diabetes is still unclear, but it appears that an increase in the secretion of amylin, *per se*, is not sufficient to form aggregates and amyloids. The heterozygous transgenic mice that produce a large amount of human amylin do not develop islet amyloids, and only some, but not all, homozygous mice develop this pancreatic pathology (Hoppener et al., 1999), which requires extrapancreatic and environmental factors such as high-glucose or high-fat feeding to occur (Hull et al., 2003; Andrikopoulos et al., 2004). These factors, which can promote the formation of amylin amyloids, include the following: (1) insulin resistance like hyperglycemia (de Koning et al., 1994), (2) the ob gene introduction (Hoppener et al., 1999), or (3) glycosylation of amylin (Kapurniotu et al., 1998). Notably, the changed ratio of amylin to insulin in plasma induced by diet or other experimental stimulations is related to the formation amylin amyloid in the pancreas (Gebre-Medhin et al., 2000).

### Future perspectives in developing amylin type peptides as a treatment for AD

Although amylin's self-aggregation property under the pathlogical conditions (Pillay and Govender, 2013) may affect its development as a drug for AD, several studies demonstrate that amylin can inhibit Aβ aggregate, as they can form the cross-interactions (Andreetto et al., 2010; Seeliger et al., 2012). Monomeric amylin and its analogs inhibit the formation of Aβ aggregation *in vitro* (Yan et al., 2007, 2013, 2014; Sellin et al., 2010; Andreetto et al., 2011). It is shown that amylin with the methylation at N-terminal region is highly soluble and inhibits the aggregation of Aβ40 (Yan et al., 2007, 2013; Sellin et al., 2010). N-terminal region of amylin is critical for inhibiting Aβ fribrillogenesis and cell toxicity through Aβ-amylin interaction (Andreetto et al., 2011). As the Aβ oligomer is a key element in the AD pathogenesis (Selkoe, 2008), that Aβ-amylin hetero-oligomers are not cytotoxic (Yan et al., 2014) may be another mechanism to reduce amyloid pathology in the brain. Rodent amylin also shows an ability to inhibit formation of fibrils from human amylin (Westermark et al., 2000), and can not induce cell apoptosis like human amylin after incubating for 48 h (Ritzel et al., 2007).

The debate of over 10 years ago on whether amylin is beneficial or harmful to the treatment of type 2 diabetes (Gebre-Medhin et al., 2000) may provide a lesson for drug development for AD. Since the clinical utility of human amylin is limited by a propensity for self-aggregation despite its activity of inhibiting appetite and regulating glucose metabolism, that limitation was overcome by the substitution of prolines at positions 25, 28 and 29 of human amylin based on rat amylin sequences (Colburn et al., 1996; Moriarty and Raleigh, 1999). This resulted in a synthetic amylinomimetic peptide, pramlintide, with improved stability and decreased potential for aggregation and pramlintide has become a potent anti-diabetic drug (Pencek et al., 2010). Pharmacokinetic studies show that the terminal half-life of amylin in rats is ~13 min and that the half-life for pramlintide in humans is ~20–45 min (Colburn et al., 1996; Young, 2005). Pramlintide has a favorable safety profile in clinical use, and only nausea is the most common tolerability-related adverse event (Aronne et al., 2007).

Since pramlintide is a relatively new drug for diabetes, there are no available data on the association between pramlintide use and AD yet. However, some medications, which are shown to influence the concentration of amylin in blood, are associated with cognitive function in humans. For example, metformin is shown to lower serum amylin concentrations in patients with type 2 diabetes (Zapecka-Dubno et al., 1999), and use of metformin may be associated with cognitive impairment (Moore et al., 2013) and increased risk of AD development (Imfeld et al., 2012). In contrast, another drug sulfonylurea does not affect serum amylin concentrations (Rachman et al., 1998), and is found not associated with the risk of AD. One animal study shows that sulfonylurea treatment reduces the AD pathology in the brain (Baraka and ElGhotny, 2010).

While many papers in the AD field have been focused on amylin's self-aggregation like Aβ (Gotz et al., 2009; DeToma et al., 2012), probably equal attention needs to be given to the potential benefits of soluble amylin or its non-amyloidgenic analogs for the AD brain. The research findings that amylin readily crosses the BBB, mediates important brain functions and mimics the Aβ structure so that they can antagonize each other are hard to ignore, taken together. Based on ours and Adler et al.,'s studies, it is hypothesized that this old foe, amylin, or its analogs may become a new friend for AD. More basic researches are needed to understand the mechanism of amylin's effects in the AD brain and probably to search for better analogs of it. Ultimately, whether amylin type peptides can be a new and novel avenue of therapeutic for AD should only be concluded through a double blind, placebo controlled clinical trial in humans.

### Conflict of interest statement

Wei Qiao Qiu and Boston University have filed a patent application for their amylin findings. The authors declare that the research was conducted in the absence of any commercial or financial relationships that could be construed as a potential conflict of interest.
